# Scalp Plexiform Neurofibrosarcoma With Intrathoracic Fibrosarcoma: A Case Report

**DOI:** 10.7759/cureus.27853

**Published:** 2022-08-10

**Authors:** Titus O Chukwuanukwu, Alvan-Emeka K Ukachukwu, Kenneth C Etukokwu, Evaristus E Afiadigwe, Akunne I Apakama, Arthur E Anyabolu, Michael E Onwukamuche

**Affiliations:** 1 Plastic and Reconstructive Surgery Unit, Department of Surgery, Nnamdi Azikiwe University Teaching Hospital, Nnewi, NGA; 2 Duke Neurosurgery/Duke Global Neurosurgery and Neurology, Duke University Medical Center, Durham, USA; 3 Cardiothoracic Surgery Unit, Department of Surgery, Nnamdi Azikiwe University Teaching Hospital, Nnewi, NGA; 4 Department of Otorhinolaryngology, Nnamdi Azikiwe University Teaching Hospital, Nnewi, NGA; 5 Department of Ophthalmology, Nnamdi Azikiwe University Teaching Hospital, Nnewi, NGA; 6 Pulmonology Unit, Department of Medicine, Nnamdi Azikiwe University Teaching Hospital, Nnewi, NGA; 7 Department of Pathology, Nnamdi Azikiwe University Teaching Hospital, Nnewi, NGA

**Keywords:** intra-thoracic metastases, neurofibrosarcoma, malignant peripheral nerve sheath tumor (mpnst), scalp plexiform neurofibroma, neurofibromatosis type 1 (nf1)

## Abstract

Neurofibromatosis type 1 (NF1) is the most common form of neurofibromatosis. It is associated with neurofibromas, gliomas, neurofibrosarcomas, and neuroendocrine and hematopoietic tumors. We present a case of scalp plexiform neurofibromatosis associated with intrathoracic fibrosarcoma.

An 18-year-old female presented with a 15-year history of plexiform scalp mass. She had multiple café-au-lait patches on her trunk and extremities and a first-degree relative with a plexiform right shoulder mass. She was managed by a multidisciplinary team of plastic and reconstructive surgeons, neurosurgeons, cardiothoracic surgeons, otorhinolaryngologists, ophthalmologists, pulmonologists, and pathologists. The histology of the excised scalp mass was that of a malignant peripheral nerve sheath tumor (neurofibrosarcoma). She subsequently developed upper chest and back pain with associated breathlessness and was found to have an intra-thoracic tumor. She had two sessions of exploratory right thoracotomy with subtotal excision of an aggressive, highly hemorrhagic, infiltrative mucinous tumor. The histology was a fibrosarcoma. The patient died a few hours following the second thoracotomy.

NF1 is associated with several tumors, among which are neurofibrosarcomas. Intra-thoracic fibrosarcoma requires aggressive surgical resection; recurrence may be delayed with radiotherapy and chemotherapy. The prognosis is however poor, and survival beyond one year is unusual. Once one tumor is found, other body systems should be evaluated for the possibility of other tumors.

## Introduction

Neurofibromatosis type 1 (NF1), also known as von Recklinghausen’s disease, accounts for over 90% of all neurofibroma cases [1,2]. It is a benign tumor of peripheral nerves with proliferating neural crest-derived Schwann cells, perineural cells, and endoneural fibroblasts [1,3,4]. Genetically, it arises from the defective NF1 gene on chromosome 17q11.2 whose protein product neurofibromin is a tumor suppressor and mutates spontaneously in 50% of cases [1,5-9]. NF1 is inherited as autosomal dominant with complete penetrance and is seen in all races and genders [3,6-8]. A variable reported incidence of 1 in 2000-4000 live births has been documented [1,6-8]; higher in Asians (57.4%) and Africans (12.8%) [2].

NF1 may be present as nodular, diffuse, or plexiform lesions in different parts of the body [2,4]. Scalp plexiform neurofibromatosis was first reported in 1906 by Helmholtz and Cushing in a 19-year-old man with generalized neurofibromatosis and a scalp mass [10,11]. They called it "elephantiasis nervorum of the scalp" and noted a predilection for the trigeminal field and temporal region [10]. NF1 may be associated with several tumors, including neurofibromas, gliomas, neurofibrosarcomas, neuroendocrine tumors (such as pheochromocytomas), and hematopoietic tumors [3,8,9,12]. They may include benign and malignant intrathoracic tumors occurring de novo or from the transformation of pre-existing neurofibromas of the vagus, phrenic, recurrent laryngeal, intercostal, or spinal nerves [3,5,7-9,12,13]. Hearing loss is rare with NF1 [1].

Few reports of neurofibromatosis, including scalp and intrathoracic neurofibromas, have been documented in our subregion [1,5-7,9,14-15]. However, to the best of our knowledge, this is the first report of a coexisting scalp plexiform neurofibroma and an intrathoracic fibrosarcoma.

## Case presentation

An 18-year-old female presented with a 15-year history of insidious scalp swelling involving the right orbito-fronto-parieto-temporal region. It was painless, firm, and multinodular, with an ulcerated summit that bled intermittently and had purulent necrotic exudate. She had difficulty lifting her head, generalized dull throbbing headaches, intermittent moderate-grade fever, right-sided visual deficit, and progressive weight loss. There was a positive history of a similar swelling on her father’s left shoulder, but no history suggestive of malignancy. The patient was abandoned by relatives in her rural community and was cared for by a self-acclaimed community social worker who used her for alms begging. She presented to our team for treatment of the infected scalp ulcer. Most of her care was borne by our hospital’s Medical Social Services Department as her caregiver eventually abandoned her.

On examination, she was chronically ill-looking, cachectic, pale, and unable to lift her head without support. Eye examination showed a 7 mm dilated sluggish right pupil, visual acuity Counting fingers on the right, right papilledema, and right mechanical ptosis and facial paresis. There was a 30 cm × 24 cm × 10 cm firm, multilobulated right orbito-fronto-parieto-temporal scalp mass with a 12 cm × 8 cm fungating sloping-edged ulcer with a bleeding necrotic floor. The surrounding scalp was inflamed and tender, and there were multiple discrete occipital and cervical lymphadenopathy. She had over ten café-au-lait patches on the trunk and extremities and multiple hyperpigmented nodules on the trunk. Examination of other body systems was normal.

Our clinical diagnosis was a huge right orbito-fronto-parieto-temporal scalp plexiform neurofibroma with septic ulceration. A cranial computed tomography (CT) scan showed a huge multilobulated mixed-density scalp mass extending from the bifrontal region to the right parieto-temporal region, involving the right upper eyelid and superior part of the right orbit, with no intracranial extension. Initial chest radiography and other metastatic screenings were negative.

**Figure 1:**
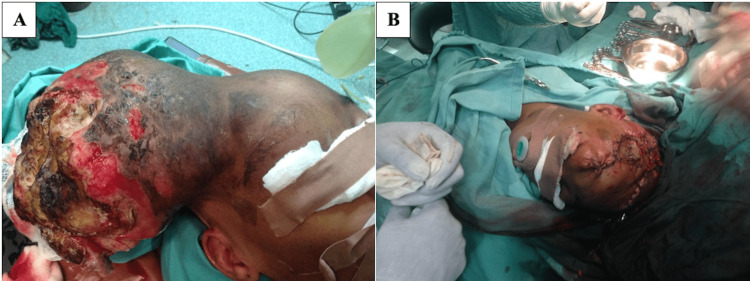
(A) Huge scalp mass with ulcerations, septic necrotic slough, purulent exudate, and inflamed surrounding scalp. (B) Near-total tumor excision with full-thickness skin graft cover achieved.

The patient was managed by a multidisciplinary team of plastic and reconstructive surgery, neurosurgery, otorhinolaryngology, cardiothoracic surgery, ophthalmology, and pulmonology units. At initial surgery, the intraoperative finding was a huge scalp mass that was soft-to-firm in consistency, fluctuant in some areas, ulcerated, with septic necrotic slough and purulent exudate, and an inflamed overlying scalp extending to the right temporal region (Figure 1A). Near-total tumor excision and full-thickness skin graft cover of the resulting scalp defect were done (Figure 1B).

**Figure 2:**
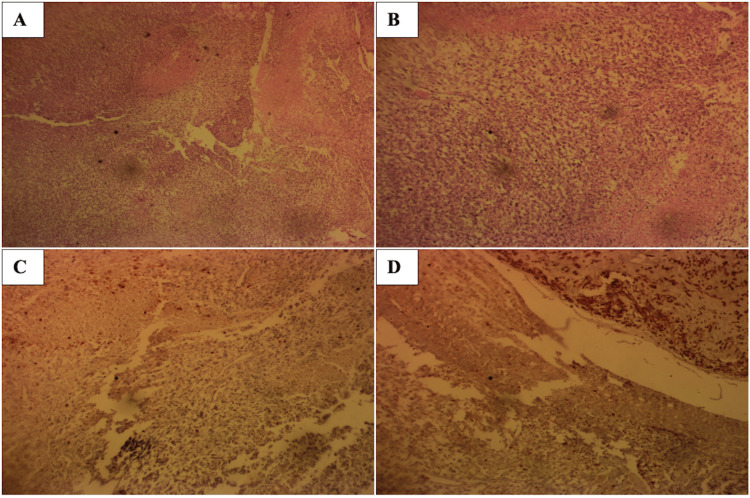
(A) H&E stain section at ×5 magnification shows a malignant mesenchymal neoplasm composed of spindle cells having large hyperchromatic nuclei, scant cytoplasm disposed in sheets separated by thin fibrocollagenous stroma, with foci of geographic necrosis. (B) H&E section of the same lesion at ×10 magnification. (C) Immunostaining for S-100 showing focal positivity, ×10 magnification. (D) Another section with immunostaining for S-100 showing focal positivity at ×10 magnification. H&E: hematoxylin and eosin.

Macroscopy of the excised tissue showed partly skin-covered multinodular tissue measuring 20 cm × 19.5 cm × 15 cm, weighing 3 kg, with a variegated cut section with multiple cysts containing gel-like material and variable-sized nodules. Microscopy was consistent with malignant mesenchymal neoplasm composed of spindle-shaped cells having large vesicular nuclei, prominent nucleoli, and scanty cytoplasm disposed in herringbone to storiform pattern and separated by thin fibro-collagenous stroma (Figure 2A-2B). There were multifocal areas of cellular and hypocellular patterns and mitoses, including abnormal variants. Immunostaining for S-100 showed focal positivity (Figure 2C-2D). The histological diagnosis was malignant peripheral nerve sheath tumor (neurofibrosarcoma).

**Figure 3:**
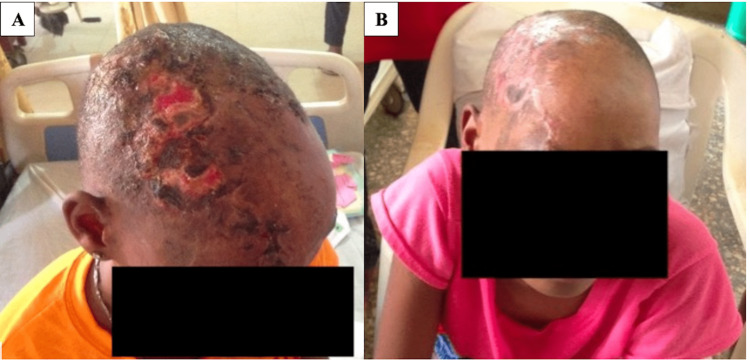
(A) Post-operative superficial wound dehiscence which recovered with serial dressing. (B) Good cosmetic outcome following complete healing. The patient had at this point developed chest pain and breathlessness. Note lean-forward sitting position.

Postoperatively, she received parenteral antibiotics and analgesics; she had superficial wound dehiscence managed with regular dressings (Figure 3A-3B). However, on the 15th postoperative day, she developed upper chest and back pain, worse with upper limb movement and with associated breathlessness. Chest examination showed elevated right hemithorax with decreased tactile and vocal fremitus, dull percussion notes, and decreased breath sounds in the right upper lung zone. The clinical diagnosis was an intraparenchymal right upper lung tumor. A chest radiograph showed well-circumscribed pleural-based masses in the right upper and mid and left upper lung zones. A high-contrast chest CT scan showed bilateral benign-looking posterior mediastinal masses with trachea compression. The differentials were neurofibroma and ganglioneuroma.

An exploratory right thoracotomy showed intra-operative findings of an irregular friable mucinous highly vascular right upper lobe mass infiltrating the right middle lobe, multiple broncho-pleural fistulas, and multiple mucinous nodules on the inner chest wall and pleura. A right upper lobectomy was done, with a right closed thoracostomy tube drain (CTTD) with an underwater seal left in situ. Intercostal block analgesia was used in the immediate postoperative period. The patient’s complaints of recurrent chest pain and breathlessness persisted after an initial improvement. She was unable to get repeat cranial and chest CT scans due to a lack of funds. Re-do exploratory right thoracotomy 27 days post-initial thoracotomy revealed an aggressive-looking highly hemorrhagic infiltrative mucinous tumor. The tumor was debulked and a right CTTD was left in situ. The patient’s clinical status, however, remained poor in the immediate postoperative period, and she suffered a cardio-pulmonary arrest about two hours post-surgery. The histology of the debulked intrathoracic tumor was fibrosarcoma.

## Discussion

The diagnosis of NF1 may be clinical or laboratory in about 90% of the cases [8]. The clinical diagnostic criteria involve two or more of the following: six or more café au lait spots, axillary, or inguinal freckling, two or more cutaneous neurofibromas, one plexiform neurofibroma, optic glioma, two or more iris Lisch nodules, characteristic bony lesions (pseudoarthrosis, sphenoid wing hypoplasia, kyphoscoliosis), and a first degree relative with NF1 [8]. Our patient fulfilled these criteria, having multiple (>6) café au lait patches, a huge scalp plexiform neurofibroma, and a first-degree relative with left shoulder plexiform neurofibroma.

Scalp plexiform neurofibromas are well reported in the literature and may arise congenitally associated with calvaria dysplasia [4,10,14,15]. Our patient had a progressively increasing scalp mass that was first noticed at three years of age with necrosis and ulceration, presumably from outgrowing its blood supply and from recurrent friction from head coverings and beddings. The patient was admitted to the hospital with an infected bleeding ulcer and anemia. Surgical excision is the hallmark of treating plexiform neurofibromas, including scalp neurofibromas [4,10,14,15], but there is a risk of residual and recurrent tumors because of the infiltrative growth pattern [4]. Our patient had near-total excision of the plexiform scalp mass with complete wound healing; she had a residual supraorbital tumor but an otherwise good cosmetic outcome.

The several tumors associated with NF1, especially intrathoracic tumors, are usually multicentric, spread by hematogenous routes, and may exhibit aggressive and metastatic potential [8,12]. The onset of pain and progressive enlargement is known to herald malignant transformation [4]. Worse prognosis is attributed to early tumor development, and lung metastases may occur shortly after presentation [8]. At presentation and initial review, our patient had no clinical or radiological evidence of an intrathoracic lesion but developed sudden-onset chest symptoms 15 days post excision of the scalp mass. Further evaluation revealed an intraparenchymal right upper lung zone tumor. The rapidity of onset and progression of this lesion was suggestive of a malignant, poorly prognostic tumor. Whole-body positron emission tomography (PET)/CT scans have been advocated for identifying malignant changes and differentiating between malignant and benign neurogenic lesions [16]. This was not done in our patients as PET is unavailable in our locality and the cost of whole-body CT was prohibitive. We were, thus, constrained to doing cranial and chest CT scans using a fee-waiver from the hospital management. At surgery, the lesion was infiltrative, highly vascularized, and hemorrhagic, confirming the initial suspicion of malignancy. Aggressive surgical extirpation is advocated with subsequent chemotherapy and radiotherapy to delay recurrence, with survival beyond one year unusual [8]. Our patient had two partial excisions of the intrathoracic lesion aimed at debulking the tumor, causing significant respiratory distress, but succumbed soon after. She was unable to commence chemotherapy or radiotherapy following the initial surgery because of financial constraints. The histologic diagnoses of scalp malignant peripheral nerve sheath tumor (neurofibrosarcoma) and intrathoracic fibrosarcoma are consistent with NF1.

## Conclusions

Neurofibromatosis type I is associated with several tumors, amongst which are neurofibrosarcomas. Pre-existing lesions can undergo sarcomatous change and exhibit metastatic potential. Intrathoracic fibrosarcoma requires aggressive surgical resection; recurrence may be delayed with radiotherapy and chemotherapy. The prognosis following malignant change is, however, poor, and survival beyond one year is unusual.
